# Reconstructing Soil Recovery from Acid Rain in Beech (*Fagus sylvatica*) Stands of the Vienna Woods as Indicated by Removal of Stemflow and Dendrochemistry

**DOI:** 10.1007/s11270-018-4065-x

**Published:** 2019-01-22

**Authors:** Selina Türtscher, Michael Grabner, Torsten W. Berger

**Affiliations:** 1 Department of Forest- and Soil Sciences, Institute of Forest Ecology, University of Natural Resources and Live Sciences (BOKU), Peter Jordan-Straße 82, 1190 Vienna, Austria; 2 Department of Material Sciences and Process Engineering (MAP), Institute of Wood Technology and Renewable Materials, University of Natural Resources and Live Sciences (BOKU), Konrad Lorenz-Straße 24, 3430 Tulln an der Donau, Austria

**Keywords:** *Fagus sylvatica*, Soil acidification, Stemflow, Long-term trend, Dendrochemistry

## Abstract

Our goal was to reconstruct soil recovery from Acid Rain based upon removal of stemflow at beech (*Fagus sylvatica*) stands of known historic and recent soil status. Fourteen beech stands in the Vienna Woods were selected in 1984 and again in 2012 to study changes in soil and foliar chemistry over time. A part of those stands had been strip cut, and to assess reversibility of soil acidification, we analyzed soils around beech stumps from different years of felling, representing the years when acidic stemflow ceased to affect the soil. Furthermore, it was hypothesized that changes of soil chemistry are reflected in the stemwood of beech. Half-decadal samples of tree cores were analyzed for Ca, Mg, K, Mn, Fe, and Al. Soil analyses indicated recovery in the top soil of the stemflow area but recovery was delayed in the between trees areas and deeper soil horizons. Differences in soil pH between proximal and distal area from beech stumps were still detectable after 30 years indicating that soils may not recover fully from acidification or do so at a rather slow rate. Stemwood contents indicated mobilization of base cations during the early 80s followed by a steady decrease thereafter. Backward reconstructions of soil pH and soil nutrients, building on regressions between recent stemwood and soil chemistry, could not be verified by measured soil data in 1984, but matched with declining cation foliar contents from 1984 to 2012. Dendrochemical reconstructions showed highest values in the 1980s, but measured soil exchangeable cation contents were clearly lower in 1984. Hence, we conclude that our reconstructions mimicked soil solution rather than soil exchanger chemistry.

## Introduction

European beech (*Fagus sylvatica*) is one of the most important and widespread tree species in Europe, most frequently distributed in Central and Western Europe. In the last decades, beech forests have often been the subject of intensive investigations of damage caused by air pollution. Emphasis has been put on changing forest soil conditions due to acidic deposition which caused soil acidification, loss of essential base cations, increased heavy metal contents and, as a consequence, generally increased stress for several tree species (Falkengren-Grerup 1989; Kazda et al. 1986; Lindebner 1990; Shortle and Smith 2015).

International attention concerning the problem of long-range transport of air pollutants started in the 1970s as a result of atmospheric transport of sulfur emissions across state and national boundaries. In Europe, sulfur (S) emissions and deposition peaked in the early 1980s reaching loads of more than 100 kg S ha^−1^ year^−1^ (Prechtel et al. 2001). As a response to improve air quality, the United Nations Economic Commission for Europe (UNECE) implemented the Convention on Long-Range Transboundary Pollution with the aim of reducing acidic emissions. Since its implementation, emissions of air pollutants across Europe have been greatly reduced over the past decades. In Austria, SO_2_ emissions were reduced by 96% from 1980 (385.000 t) to 2015 (14.900 t) (Umweltbundesamt 2002, 2017). In order to assess the achievements of environmental protection measures, chemical comparison of forest soils in the Vienna Woods (Austria) between 1984 and 2012 indicated an early stage of reversal of the adverse effects of acidic deposition in the top soil of the infiltration zone of stemflow near the base of beech stems (Berger et al. 2016; Türtscher et al. 2017).

The resampling approach enables the study of reversibility of soil acidification between two specific soil sampling dates. A major limitation in determining changes of soil chemical properties is the absence of a historical perspective on soil chemistry and a lack of soil data prior to the accelerated increase of acidic deposition in the 1970s (Bondietti et al. 1990; Shortle et al. 2017). However, elemental analysis of dated tree rings maybe a useful tool for retrospective monitoring of atmospheric deposition and responses of forest ecosystems to altered chemical environments (Guyette et al. 1992; Watmough 1997).

Dendrochemistry, the interpretation of chemical data from dated tree rings, has been widely used to reconstruct atmospheric pollution and associated changes in soil chemistry (Berger et al. 2004; Chen et al. 2010; Guyette et al. 1992; Kuang et al. 2008), as well as for monitoring sulfur and nitrogen deposition (Penninckx et al. 1999; Struis et al. 2008), loss of base cations (Tomlinson 2003), and heavy metal contamination (Baes and McLaughlin 1984). A basic assumption in dendrochemistry is that the element content of each tree ring at least partly reflects the environmental conditions during the year of wood formation (Bondietti and McLaughlin 1992). As a consequence, tree-ring variables have been used for several years to investigate soil acidification and related changes in soil chemistry. Several authors proposed that the radial variations in wood elemental contents might reflect corresponding changes in soil pH values (Berger et al. 2004; Chen et al. 2010; Kuang et al. 2008). Guyette et al. (1992) reconstructed soil pH from Mn contents in tree rings of eastern red cedar on sites with different levels of SO_2_ deposition and considered Mn contents in tree rings as a useful tool for examining long-term changes in soil pH due to acidic deposition. Manganese is an adequate marker of environmental changes, since it has a low mobilization factor and is only moderately affected by leaching (Guyette et al. 1992). Other authors reported that acidification treatments caused base cation mobilization followed by depletion that was detectable in sapwood xylem after 8 years of treatment (DeWalle et al. 1999). In addition, liming experiments might be useful to study response of trees to soil acidification even though increased soil acidification due to acidic deposition is not simply the opposite of liming. DeWalle et al. (1995) showed that Ca, Mg, and Mn contents were significantly affected by liming causing Ca and Mg to increase while Mn contents decreased due to the treatments. The use of element ratios as indicators of the effects of atmospheric deposition was found to be more pertinent as they give a relative view of change of one element against another and the use of ratios normalizes for fluctuations in cation contents (Bondietti et al. 1989). Elemental ratios of Ca/Mg and Ca/Mn in increment cores from *Abies fabri* were the best predictors of soil chemistry and effective to reconstruct the long-term changes of soil pH in China (Chen et al. 2010). However, results of different studies were not always consistent with each other, and the use of tree rings as indicators of environmental conditions may have some limitations, especially, when linking tree-ring chemical patterns to changes in soil chemistry. Limiting factors include radial and vertical translocations of elements, variation in mechanisms of element uptake, and variability among tree species (see review by DeWalle et al. 1991; references therein; Lepp 1975; McClenahen et al. 1989). Beech, a diffuse-porous to semi-ring porous species, lacking typical heartwood, is assumed less suitable for dendrochemical monitoring due to radial translocation of elements and, therefore, dendrochemical studies in beech trees are very rare (Hagemeyer 1993; Penninckx et al. 2001).

The chemistry of beech forest soils is characterized by a distinct heterogeneity caused by spatial patterns of throughfall and stemflow. Stemflow of beech represents a high input of water and elements and has been used to demonstrate the effects of acidic deposition on the soil near beech stems (Chang and Matzner 2000; Falkengren-Grerup 1989; Kazda and Glatzel 1984; Lindebner 1990). Enhanced soil acidification around beech stems was recorded in the Solling area in Germany (Koch and Matzner 1993) and in the Vienna Woods (Rampazzo and Blum 1992; Sonderegger 1981). Berger and Muras (2016) hypothesized that focusing on the spatial heterogeneity of soil chemistry related to the distance from beech stems enables the study of recovery of differently polluted soil, since the stemflow area near the base of the stem received much higher loads than the between trees area in the past. To assess reversibility of soil acidification, Falkengren-Grerup and Björk (1991) analyzed soils around beech stumps from different years of felling, representing the years when stemflow ceased to affect the soil. The authors observed a reduction in soil acidity by 50% after felling, which was most pronounced during the initial 15 years. Removing stemflow water experimentally showed a significant increase of soil pH close to stems in Swedish beech forests, although recovery was not completed after 8 years (Matschonat and Falkengren-Grerup 2000).

We compared soil chemical data between 1984 and 2012 for 97 old-growth beech stands in the Vienna Woods in previous studies (Berger et al. 2016; Türtscher et al. 2017). However, retrospective time series of soil chemical changes, especially before the onset of acidic deposition, are rare. Hence, in the current study, we selected 14 of these 97 sites of which a part has been strip cut in the meantime. Thus the cleared area of each site provided stumps from different years of felling, representing the years when stemflow ceased to affect the soil. Elemental contents in dated tree rings of the adjacent beech stand of each site were used to evaluate the historical response of forest soils to atmospheric deposition. We hypothesized that changing environmental conditions are reflected in soil and wood chemistry and addressed the following questions:

1. Will the soils within the stemflow area of beech approach the same chemical status of the between trees area after removal of stemflow?
2. Can the rate of reversibility of soil acidification be estimated using soil pH changes as a function of time since felling?
3. Are changes of soil chemical properties over the last seven decades reflected in the stemwood of beech?
4. Are beech xylem contents or their ratios related with measured soil pH values or soil nutrient contents?
5. Is dendrochemistry a useful tool for reconstructing soil pH and soil nutrient changes in the studied beech stands and can the data be verified by measured soil parameters in 1984?

## Materials and Methods

### Study Area and Study Sites

**Fig. 1:**
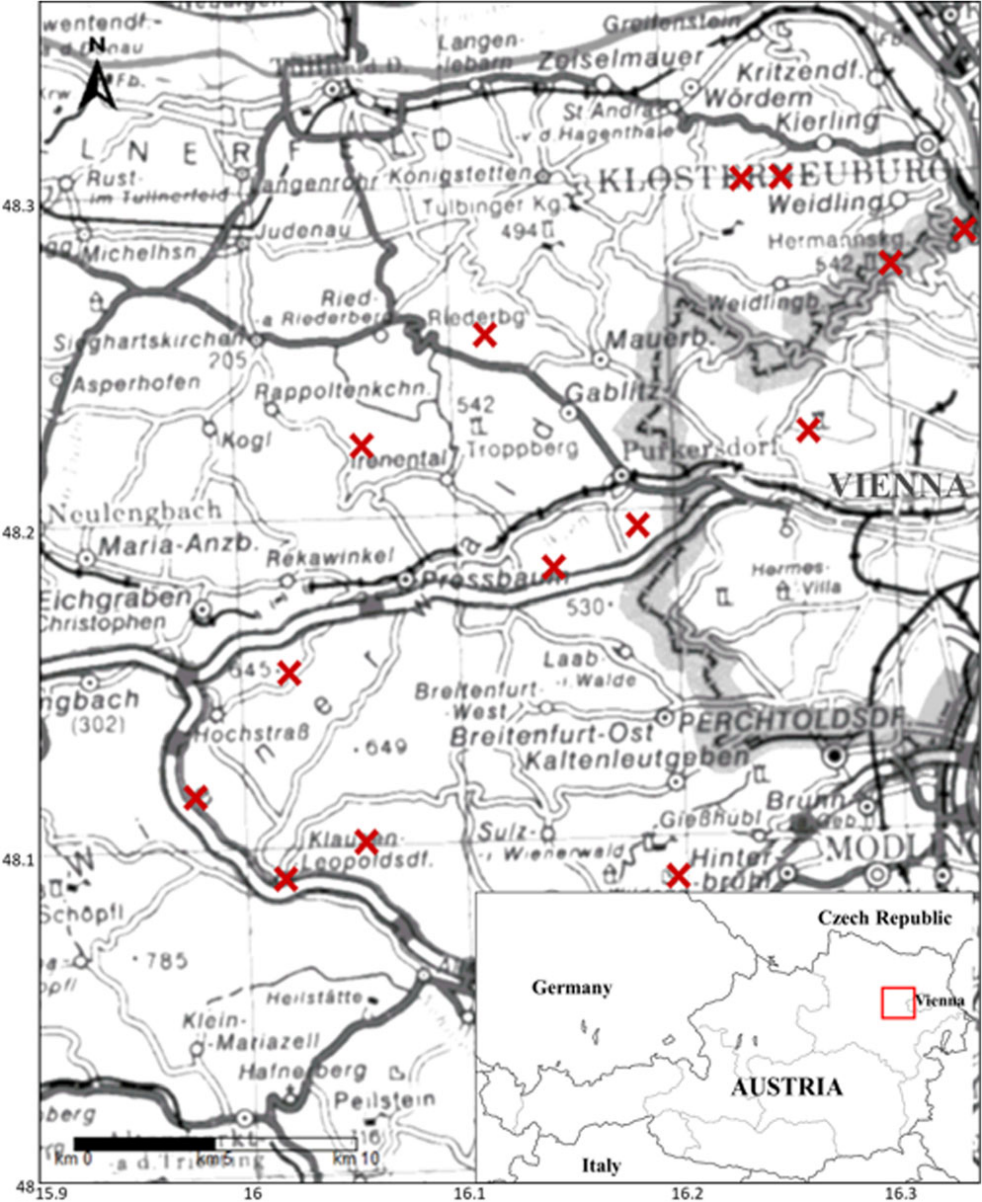
Location of the 14 study sites in the Vienna Woods

The 14 study sites are located in two federal states, Lower Austria and Vienna, within the so-called Vienna Woods, a forested highland that forms the foothills of the Northern Limestone Alps. The total area of the Vienna Woods is about 125.000 ha, situated north, west, and south of the City of Vienna (Austria). Elevations range from about 180 m to over 800 m a.s.l. The mean annual precipitation varies between 600 and 900 mm, the mean annual temperature is 8–9 °C, and the two main wind directions are west winds all over the year and south-east winds mostly in fall and winter. The major part of the Vienna Woods is forested land. Beech (*Fagus sylvatica*) is the main tree species, representing 50% of the standing timber volume. Geologically, two bedrock types can be distinguished: the bedrock of the northern, major part of the Vienna Woods is Flysch and the southern, much smaller part is limestone (Plöchinger and Prey 1974; Rieder 2002). All 14 study sites were selected in pure old-growth beech stands on Flysch in the northern and western part of the Vienna Woods (Fig. 1). Flysch consists mainly of old tertiary and mesozoic sandstones and clayey marls. Nutrient release from this bedrock is high, and therefore, the prevalent humus forms are mull to intermediate types between mull and moder, indicating quick turnover of the forest litter layer (usually less than 2 cm thickness) and nutrient-rich soils. All soils of these study sites were classified as pseudogley (Scheffer and Schachtschabel 2010; WRB classification: endostagnic cambisol), since horizons with a high fraction of fine material (loam to clay) cause temporary waterlogging (stagnation zone at approximately 40–50 cm soil depth).

**Fig. 2:**
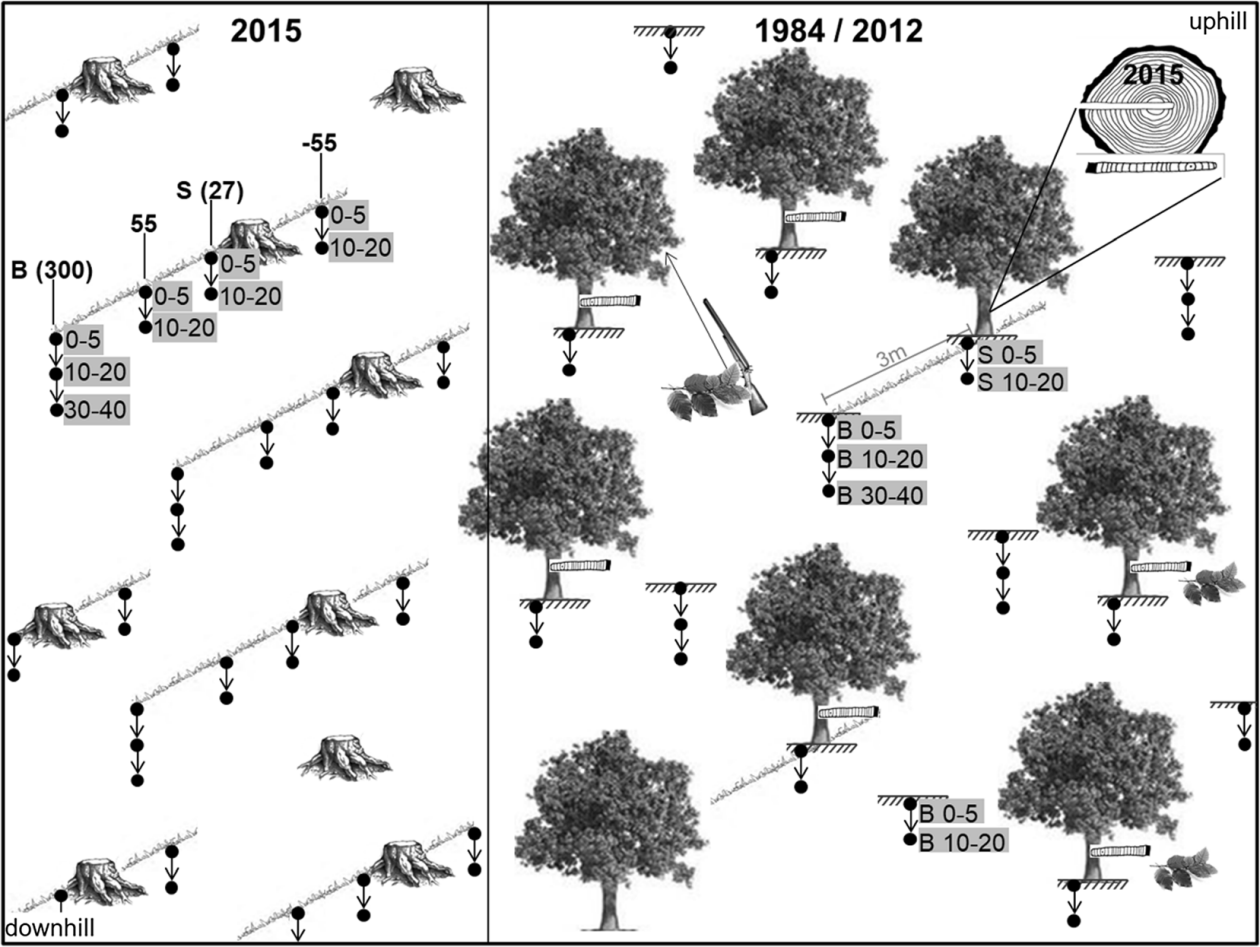
Schematic map of one site, existing of a strip cut plot (sampled in 2015) and an adjacent beech stand (sampled in 1984 and 2012), and measurements performed. Note that soil samples in 10–20 cm depth were collected only in 2012

In the early 1980s, 152 pure old-growth beech stands were selected for soil sampling by Lindebner (1990). At that time, all stands were older than 80 years and had a stand density index of > 0.8. In 2012, 97 of the 152 beech stands still existed for repeated sampling (Berger et al. 2016; Türtscher et al. 2017). In both years (1984 and 2012), soil samples were taken from the infiltration zone of stemflow near the base of the stem (S) and from the between trees area at least 3 m away from the stems (B) in different soil depths. We selected 14 of these 97 beech stands of which a part has been strip cut in the meantime (Fig. 2). The cleared area of each site (strip cut plot) provided stumps (adjacent to the original stand of each site) from different years of felling, representing the years when stemflow ceased to affect the soil.

### Sampling and Analytical Methods

#### Soils

The method of soil sampling at the 97 beech stands in the years 1984 (summer) and 2012 (spring) is given in details by Berger et al. (2016) and Türtscher et al. (2017) but bears repeating at this point: seven top mineral soil samples (0–5 cm depth) per site (see Fig. 2; beech stand) from the infiltration zone of stemflow (20 cm downhill from the base of the stem, one sample per beech tree, S 0–5) and from the between trees area (at least 3 m away from beech stems, B 0–5) were taken with a cylinder (diameter 50 mm; height 50 mm). Additional soil samples were taken with a soil auger (diameter 20 mm, half open steel pipe) from 10 to 20 cm depth (S 10–20; seven replications) only for the year 2012. In each case, all seven replicated samples per stand were pooled before chemical analysis. Furthermore, the 20-mm soil auger was used for collecting soil samples from 10–20 cm (B 10–20, only in 2012) and from 30–40 cm depth (B 30–40; both years) and seven and four replications per horizon, respectively, were pooled before chemical analysis.

In April 2015, 14 of these 97 old-growth beech stands were selected of which a part has been strip cut (see Fig. 2; strip cut plot). Those sites provided beech stumps of known felling time. A total of 98 stumps were studied, seven stumps at each strip cut plot. Top mineral soil samples (0–5 cm depth) were taken with a cylinder (diameter 50 mm; height 50 mm) at each of the following four distances from seven beech stumps per site: at 55 cm uphill (further labeled − 55), and at 27 (infiltration zone of stemflow, S 0–5), 55, and 300 (between trees area, B 0–5) cm downhill from the stump. Again, in each case, all seven replicated samples per strip cut plot were pooled before chemical analysis. Additional soil samples were taken at the same distances with a soil auger (diameter 20 mm, half open steel pipe) from 10–20 cm soil depth (seven replications per plot were pooled). Moreover, we used the 20 mm diameter auger for sampling soil in 30–40 cm soil depth from the between trees area (B 30–40, seven samples adjacent to the 0–5 and below the 10–20 cm collection were pooled).

Soil chemical parameters were determined by standard procedure as suggested by Blum et al. (1989) for the standardization of Austrian soil surveys (more details are given by Berger et al. 2016): In 1984, mineral soil (< 2 mm) was analyzed for total content of C (Wösthoff Carmhomat ADG 8, Germany) and S (LECO SC 132, USA) according to ÖNORM L1080 and total N (Kjeldahl method, 2300 Kjeltec Analyzer Unit, Tecator, Sweden) according to ÖNORM L1082. In 2012 and 2015, total C, S, and N were analyzed by LECO SC 444 (USA, ÖNORM L1080). Organic C was calculated total C minus C_CaCO3_ (Scheibler method: reaction of carbonates with HCl and volumetric determination of emerging CO_2_ according to ÖNORM L1084). Calcium, Mg, and K were measured as exchangeable cations (1 M ammonium acetate extract at pH 7, ÖNORM L1086) by graphite furnace atomic absorption spectrometry (GF-AAS, Perkin Elmer 3030, USA) in the 1984 samples and by inductive coupled plasma optical emission spectrometry (ICP-OES, Optima 3000 XL, Perkin Elmer, USA) in the 2012 and 2015 samples. Soil acidity was measured as pH with a glass Ag/AgCl combination electrode with KCl reference electrode: 10 g soil was mixed with 25 ml of 0.1 M KCl or deionized H_2_O, stirred, and the pH was measured next morning 30 min after stirring again (ÖNORM L1083).

#### Foliage

In late August/early September 1984 and in early September 2012, leaf samples of beech were collected with a shot gun from the upper crown of three trees per site (see Fig. 2; beech stand). All subsamples per stand were pooled before analysis, yielding approximately 60–100 leaves.

Foliage samples were dried at 105 °C and ground. Total contents of N and S were analyzed as described for the soil samples above. Calcium, Mg, and K were measured as total contents after digestion with HNO_3_/HClO_4_ (ÖNORM L1085) by GF-AAS (1984 samples) and ICP-OES (2012 samples), respectively.

#### Tree Rings (Dendrochemistry)

At each of the 14 beech stands, the same seven healthy dominant beech trees of which soil samples were taken from the infiltration zone of stemflow near the base of the beech stem in 1984 and 2012 were selected for coring. In April 2015, one increment core of 5 mm diameter was removed from each stem at approximately 1.3 m aboveground. Cores were stored in plastic straws in the field, transferred to the freezer (− 18 °C) at the laboratory, and kept there until analysis. The diameter of each tree was measured where the cores were extracted for transformation of ring width to basal area increment (BAI). Growth was calculated using the following formula: BAI = *π* * (*R*^2^_*n*_ − *R*^2^_*n* − 1_), where *R* is the tree radius (distance from the pith to the other boundary of the ring formed in year *n*) and *n* is the year of tree-ring formation.

Cores were sliced horizontally (i.e., lengthwise) while still moist with a stainless steel blade to reveal annual growth rings. Cores were digitally scanned to record the image of the incremental rings. Annual growth rings of each core were dated by the WinDENDRO system (Regent Instruments Inc., Canada) and ring widths were measured at an accuracy of 0.001 mm. In order to verify that any missing or double rings were accounted for, each individual tree chronology was compared to its reference chronology based upon the site mean (seven beech trees per site). Out of 98 beech cores (14 sites × 7 trees), only six increment cores (from four sites) could not be matched with the reference chronology (site mean), which were excluded for further analysis. The growth patterns of individual trees per site were very similar, but differences between sites could partly be recorded.

Thereafter, the cores were split into 5-year increments using a stainless steel blade. According to Smith and Shortle (1996), investigations based on a single tree or core should be discouraged. Therefore, 14 recent half-decadal samples (1945–1949 to 2010–2014) of all seven trees per site were pooled and analyzed for Ca, Mg, K, Mn, Fe, and Al.

As mentioned above, beech is a diffuse-porous to semi-ring porous species, lacking typical heartwood. In comparison to other hardwood species, beech does not always form heartwood (facultative) and if it does so, heartwood develops rather late at a high age. However, since all beech trees in this study were older than 80 years in 1984, we can assume that most of the trees did form facultative heartwood, which would reduce radial movement of elements across rings.

Two different methods for the extraction of major inorganic cations were used for elemental analysis. First, a microwave digestion system (Mars 6, CEM Corporation, USA) was used to digest wood samples. Approximately 200 mg of wood chips was placed into the digestion vessels and mixed with 8 ml concentrated HNO_3_ (65%) and 2 ml high purity H_2_O_2_ (30%). After microwave digestion at 180 °C, samples were cooled and poured through a funnel with black ribbon (45 μm) filter paper into a 100 ml volumetric flask and brought to volume with deionized water. Calcium, Mg, K, Mn, Fe, and Al were analyzed by inductive coupled plasma optical emission spectrometry (ICP-OES, Optima 3000 XL, Perkin Elmer, USA). For the second method, wood samples were frozen and thawed (three times) in 0.01 M HCl. One hundred milligrams of wood chips was placed in a plastic sample vessel to which 24 ml of 0.01 M HCl was added. The samples were frozen at − 20 °C and thawed at room temperature, repeating the process two more times to ensure cell rupture and cell wall penetration to release all ionically bound base cations (Minocha and Shortle 1993; Shortle et al. 1997). The extract was then filtered and contents of Ca, Mg, K, Mn, Fe, and Al were determined by inductive coupled plasma optical emission spectrometry (ICP-OES, Optima 3000 XL, Perkin Elmer, USA).

**Fig. 3:**
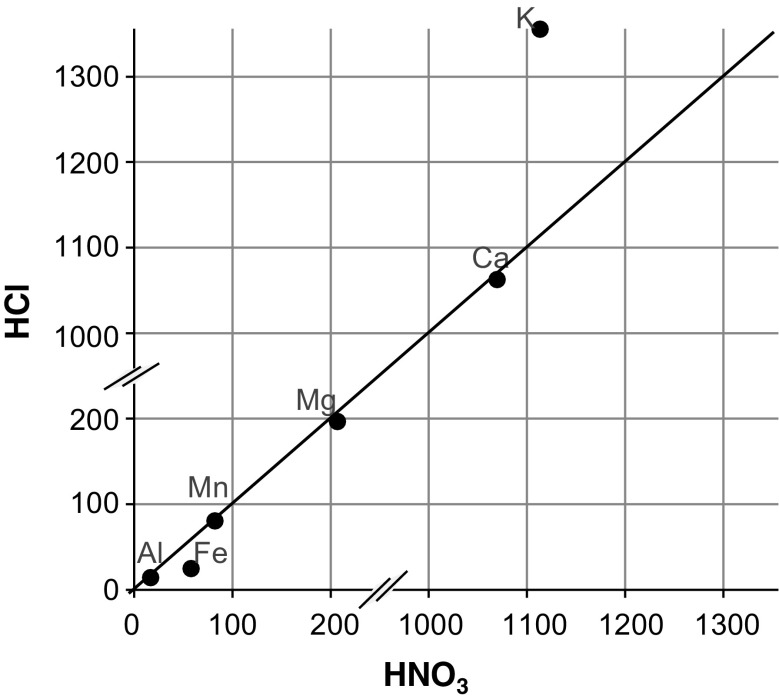
Relationship between the extraction methods HCl (plus freezing-thawing cycles) and HNO_3_ (total digestion plus H_2_O_2_) for each element measured in increment cores (average of all 14 5-year segments in μg g^−1^) of 14 beech stands in the Vienna Woods in 2015

Minocha and Shortle (1993) concluded that the extraction by freezing-thawing (FT) gives comparable results to acid digestion and there was an excellent agreement between the FT and the standard wet digestion method for the analysis of Ca, Mg, and Mn for spruce and oak wood, although higher levels of K were consistently obtained by FT over wet digestion. We compared both methods (see Fig. 3) and Ca, Mg, Mn, and Al produced similar results as well. Therefore, we only show the results obtained by microwave digestion (HNO_3_/H_2_O_2_) throughout this paper.

### Calculation of Soil pH Values and Soil Nutrients from Tree-Ring Chemistry

According to the method described by Berger et al. (2004) for spruce trees in pure spruce and mixed beech-spruce stands, we performed linear correlations for all beech stemwood cation contents and their ratios (log_10_-transformed) of the last half-decade (2010–2014) with recent soil pH data (2012) at different depths and distances from the stem: top soil in the stemflow area (S 0–5) and between trees area (B 0–5), between trees area in 30–40 cm soil depth (B 30–40). In addition, linear correlations were performed for all element (log_10_) stemwood combinations of the half-decade 1980–1984 with the 1984 soil data at the same depths and distances from the stem. Only those parameters (cation contents and/or elemental ratios) were used for further analyses for which bivariate correlations showed significant results in both years (1984 and 2012), roughly indicating that environmental processes have not changed over time. In a second step, those parameters for which bivariate correlations showed significant results were used as input variables for the performance of stepwise regressions to select the best predictor of soil pH at different depths. Finally, based on those parameters in the tree rings (2010–2014), soil pH values were reconstructed backwards by the regression equations. This means, pH values were reconstructed by regression equations based on stemwood contents/ratios of 2010–2014 and measured soil pH values of 2012, enabling a validation of the model using the soil pH values measured in 1984.

Furthermore, exchangeable soil Ca was reconstructed using the same method as described above: linear correlations for all beech stemwood cation contents and their ratios (log_10_-transformed) of the half-decade 1980–1984 and 2010–2014 with actually observed exchangeable soil Ca contents in 1984 and 2012 were obtained and stepwise regressions were performed to select the best indicator of exchangeable soil Ca contents. Calcium contents were reconstructed backwards by regression equations based on stemwood contents/ratios of 2010–2014 and measured exchangeable soil Ca contents of 2012.

## Results

### Soils

**Fig. 4:**
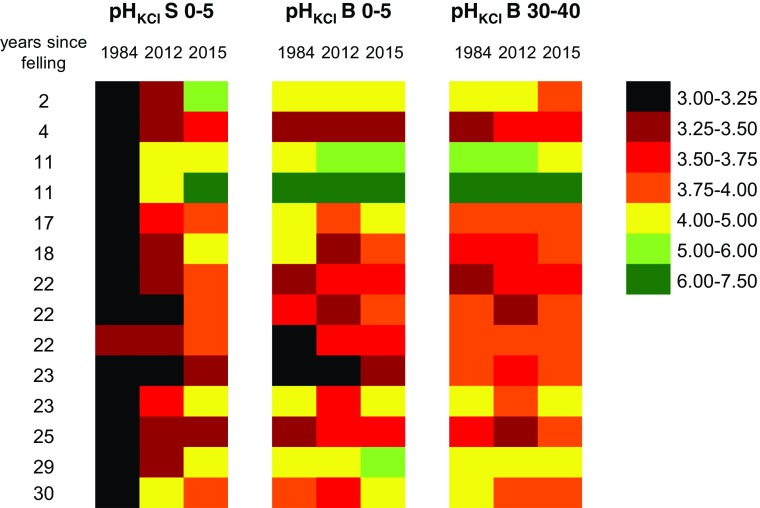
Soil pH (KCl), sorted by classes, the number of years since felling and year of sampling (1984 + 2012: soil sampling in 14 beech stands; 2015: soil sampling at the strip cut plots from beech stumps at the same 14 sites in the stemfow area in the stemflow area in 0–5 cm soil depth (S 0–5) and in the between trees area in 0–5 and 30–40 cm soil depth (B 0–5, B 30–40), respectively

**Fig. 5:**
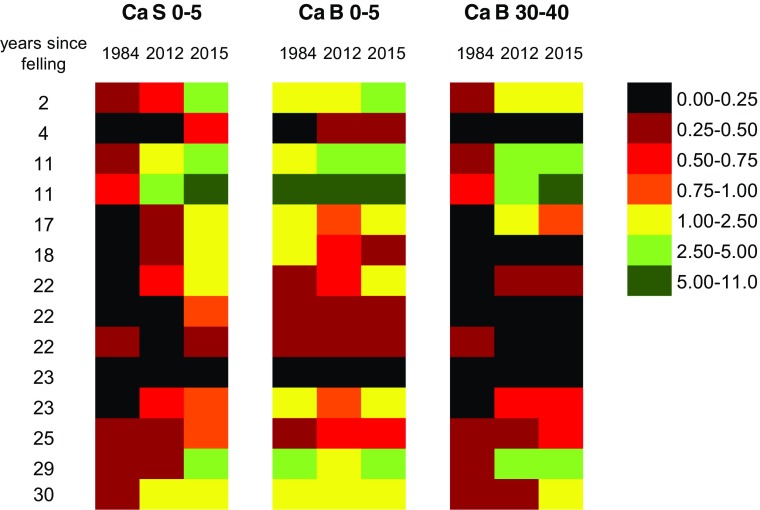
Exchangeable soil Ca (mg g^−1^), sorted by classes, the number of years since felling and year of sampling (1984 + 2012: soil sampling at 14 beech stands; 2015: soil sampling at the strip cut plots from beech stumps at the same 14 sites) in the stemflow area in 0–5 cm soil depth (S 0–5) and in the between trees area in 0–5 and 30–40 cm soil depth (B 0–5, B 30–40), respectively

To assess the recovery of the soil from the influence of stemflow water, changes in soil pH and exchangeable Ca at beech stands in 1984 and 2012 as well as at the strip cut plots (2015) were tested for different depths and distances (Figs. 4 and 5). Soil pH (KCl) within the stemflow area (S 0–5) increased at almost all (11 out of 14) stands from 1984 to 2012, whereas pH values in the between trees area have stabilized (5 out of 14 stands) or just slightly changed (both directions) in the top layers (B 0–5) and partly (5 out of 14 stands) continued to acidify in the deeper soil (B 30–40; Fig. 4). Despite distinct signs of soil recovery in the stemflow area, pH (KCl) was still lower at two thirds (9 out of 14 stands) of the beech stands compared to the between trees area (B 0–5) in 2012. At the strip cut plots (2015), soil pHs within the stemflow area (S 0–5) were higher at 11 out of 14 sites compared to the adjacent beech stands in 2012. In the between trees area (B 0–5 and B 30–40), most of the pHs were higher or in the same classes at the strip cut plots compared to the stands (2012).

Exchangeable Ca increased markedly (0.28 mg g^−1^ vs. 0.80 mg g^−1^; *p* < 0.033; data are mean values) in the stemflow area from 1984 to 2012. Trends of a Ca increase at B 0–5 from 1984 to 2012 (1.44 mg g^−1^ vs. 1.55 mg g^−1^) and from S to B at the strip cut plots (up to 2.00 mg g^−1^) were not significant (Table 1; Fig. 5). In 2012, exchangeable Ca contents decreased with soil depth from B 0–5 to B 30–40 at 9 out of 14 stands (Fig. 5). Calcium contents within the stemflow area (S 0–5) increased at 11 out of 14 strip cut plots (2015) compared to the adjacent beech stands (2012). This trend (same class or slight increase) was visible for B 0–5 and B 30–40 from 2012 to 2015 as well.

**Table 1:** Mean soil pH, mean contents of C_org_, N_tot_ and S_tot_ (mg g^-1^) and of exchangeable Ca, Mg and K (mg g^-1^) in different soil depths and distances from the stump at the strip cut plots in 2015 (-55 (uphill) and 27, 55 and 300 cm (downhill)) and from the stem at the adjacent beech stands (S: stemflow area; B: between trees area) in 1984 and 2012. Mean contents of total N, S, Ca, Mg and K (mg g^-1^) are given for fresh beech foliage in 1984 and 2012

Parameter	Soil																	Foliage			
	Depth (cm)	Strip cut plots (2015)								Beech stands											
		distance (cm) from stump								S				B							
		-55		27 (S)		55		300 (B)		1984		2012		1984		2012		1984		2012	
***pH***_***H2O***_	0-5	5.35		5.22		5.14		5.28		4.16	*a*	4.83	*b*	5.18		5.36					
	10-20	5.21		4.98		5.00		5.16				4.89				5.42					
	30-40							5.24						5.43	*a*	5.66	*b*				
***pH***_***KCl***_	0-5	4.34		4.22		4.15		4.30		3.09	*a*	3.55	*b*	4.10		4.06					
	10-20	4.11	*b*	3.95	*a*	3.98	*ab*	4.13	*b*			3.69				4.06					
	30-40							4.21						4.27		4.26					
***C***_***org***_	0-5	51.95	*ab*	66.45	*b*	60.64	*b*	44.08	*a*	105.94	*b*	60.80	*a*	45.16		50.34					
	10-20	23.44		25.27		25.75		22.36				26.46				23.76					
	30-40							9.57						13.87		14.03					
***N***_***tot***_	0-5	3.23	*ab*	3.91	*b*	3.70	*b*	2.94	*a*	6.19	*b*	4.17	*a*	2.56		3.37		23.03		22.44	
	10-20	1.64		1.69		1.74		1.61				1.82				1.69					
	30-40							0.81						0.86		1.06					
***S***_***tot***_	0-5	0.43	*ab*	0.57	*c*	0.51	*bc*	0.36	*a*	0.79	*b*	0.53	*a*	0.21	*a*	0.42	*b*	1.51		1.53	
	10-20	0.21		0.23		0.24		0.21				0.22				0.18					
	30-40							0.10								0.17					
***Ca***	0-5	2.05		1.96		1.83		2.00		0.28	*a*	0.80	*b*	1.44		1.55		12.22	*b*	9.56	*a*
	10-20	1.41		1.16		1.15		1.56				0.94				1.67					
	30-40							1.64						1.89	*b*	1.14	*a*				
***Mg***	0-5	0.16		0.16		0.16		0.14		0.06		0.08		0.11		0.13		2.00	*b*	1.24	*a*
	10-20	0.09		0.06		0.07		0.08				0.06				0.10					
	30-40							0.08						0.14		0.08					
***K***	0-5	0.15	*a*	0.21	*b*	0.19	*ab*	0.14	*a*	0.28	*b*	0.13	*a*	0.15	*b*	0.10	*a*	12.78	*b*	6.64	*a*
	10-20	0.09		0.13		0.13		0.09				0.08				0.08					
	30-40							0.13						0.10		0.07					

A repeated measures ANOVA was performed for each parameter separately and results of (multiple) Bonferroni corrected paired comparison tests between distances per depth (strip cut plots), between the years 1984 and 2012 per distance and depth (beech stands) and between the years (foliage) are given only, if differences were significant (different letters indicate significant differences, p < 0.05; *a* represent the lowest means)

Top soil pH within the stemflow area increased significantly by 0.7 and 0.5 units in both H_2_O and KCl extracts from 1984 to 2012, respectively (Table 1). Exchangeable Ca (significantly) and Mg (trend) increased in the stemflow area and to a lower extent in the top soil of the between trees area. Contents of C, N, S, and K decreased significantly in the stemflow area from 1984 to 2012, suggesting that mineralization rates of organic matter increased due to more favorable soil conditions.

Mean soil pH, contents of *C*_org_, *N*_tot_, and *S*_tot_ (mg g^−1^) and of exchangeable Ca, Mg, and K (mg g^−1^) of top soil (0–5 cm soil depth) at − 55 (uphill) and 27, 55, and 300 cm distance to the base of beech stumps are presented in Table 1. Although soil pH in both H_2_O- and KCl extracts decreased towards the stump, no significant impact of distance on pH patterns was recorded (Table 1; letters are only given, if differences are significant). However, effects of stemflow-induced acidification were still recognizable, even after removing of beech stems. Contents of S and N were significantly higher (as indicated by different letters in Table 1) and contents of exchangeable Ca tended to be lower near the base of the beech stumps (S) than in the between trees area (B). Although stemflow gradients still existed for most parameters after removal of stemflow by felling beech trees, the difference between the distances from the stumps was not significant for pH (H_2_O and KCl) and exchangeable contents of Ca and Mg. Patterns of soil pH with distance from the stump were relatively the same in 10–20 soil depth; however, soil pH measured in KCl was significantly different at the different distances (see different letters in Table 1). Soil pH in 30–40 cm soil depth in the between trees area tended to be lower compared to the top soil (0–5 cm soil depth) in both H_2_O- and KCl extracts. Contents of C, N, and S as well as exchangeable Ca, Mg, and K did not differ significantly at the different distances from the stump in 10–20 cm soil depth. However, a significant decline with soil depth in the between trees area was observed for C, N, and S contents.

**Fig. 6:**
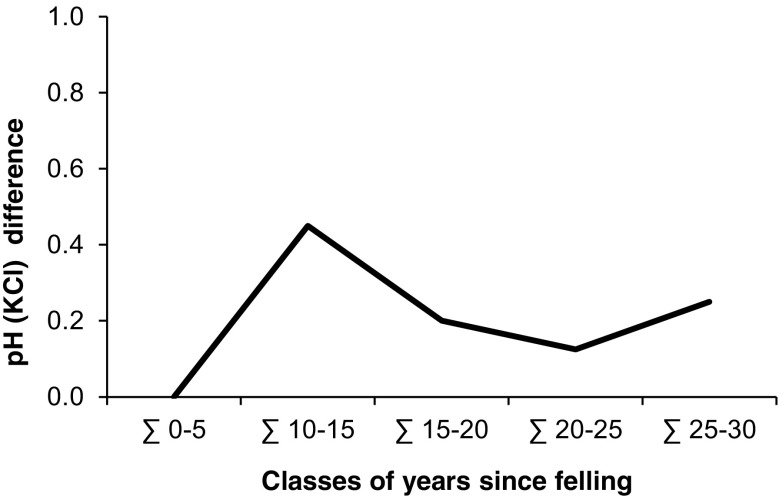
Difference in pH (KCl) between distal (300) and proximal (27) soil samples in 10–20 cm soil depth along a downhill slope gradient from beech stumps at 14 sites in the Vienna Woods in 2015, related to classes of years since felling

The hypothesis, that the longer the time that had elapsed since felling, the smaller the difference between proximal and distal values would be, seemed to be only partly validated. As shown in Fig. 6, the decreasing pH (KCl) difference in 10–20 cm soil depth implies that the soil partly returned to the overall acidity of the plot since the difference between distal (300 cm away from the stumps) and proximal (27 cm away from the stumps) area became smaller with increasing age of the stumps. However, the largest difference in pH (KCl) was not found in the youngest year class but rather in the second class (10–15 years since felling).

### Tree Rings (Dendrochemistry)

#### Element Contents and Ratios in Stemwood

**Fig. 7:**
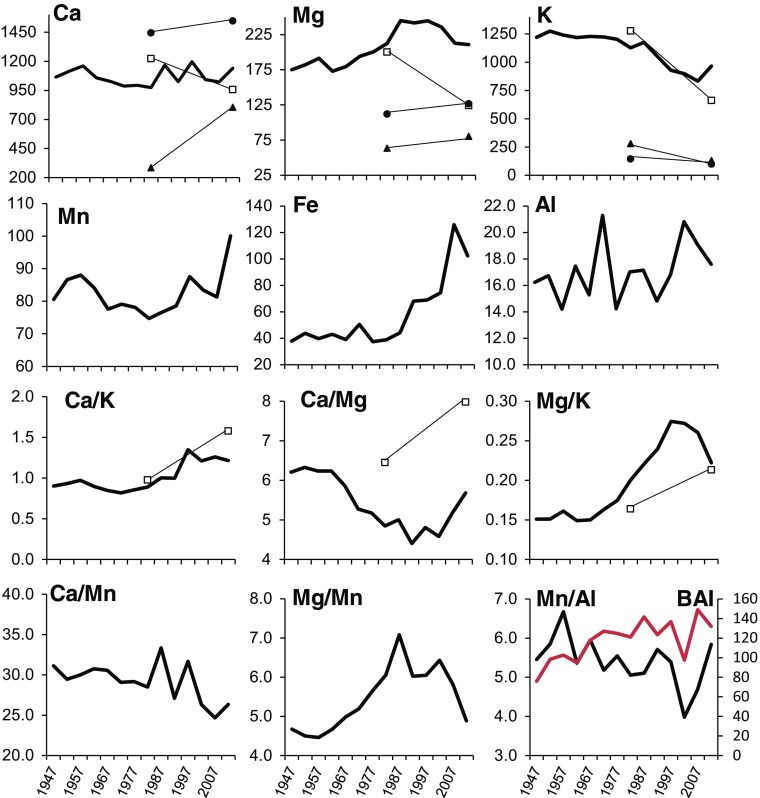
Mean Ca, Mg, K, Mn, Fe, and Al (μg g^−1^) stemwood contents, their ratios (Ca/K, Ca/Mg, Mg/K, Ca/Mn, Mg/Mn, and Mn/Al), and mean basal area increment (BAI; cm^2^) of beech at 14 beech stands in the Vienna Woods from 1945–1949 to 2010–2014 (every second 5-year segment is labeled by the year of the midperiod). Measured data of exchangeable soil Ca, Mg, and K (μg g^−1^) are plotted for the years 1984 and 2012 for 0–5 cm depth in the stemflow area S 0–5 (filled triangle) and between trees area B 0–5 (filled circle) and connected via a trend line. Similarly, measured fresh beech foliage data of Ca, Mg, K and the corresponding ratio combinations (μg g^−1^ divided by 10) are given for the years 1984 and 2012 (open square)

Mean Ca, Mg, K, Mn, Fe, and Al stemwood contents (μg g^−1^) and their ratios of beech from 1945–1949 to 2010–2014 are plotted in Fig. 7. The half-decadal contents are quite variable across the range of elements analyzed. Four patterns may be distinguished: an increase in Mn and Fe since the late 1970s/early 1980s, a decreasing trend of Mg after the late 1980s, no clear trend for Ca and Al, and a decrease in potassium (K). Mean half-decadal ratios of beech stemwood contents also showed different patterns over time. It seemed that all patterns had several peaks during the observed time span. However, we would expect a steady decline of cation stemwood contents from older to younger wood due to a decreasing cation binding capacity with increasing distance from the pith (Momoshima and Bondietti 1990). Trends of declining cation stemwood contents were recorded only for K. Overall, there was no evidence for a sharp heartwood/sapwood transition, contrary to other tree species, where a minimum or maximum of nutrient contents at the heartwood/sapwood boundary region is usually found (Penninckx et al. 2001). BAI (basal area increment) of beech increased over time (Fig. 7).

### Reconstruction of Soil pH and Soil Ca

The results of linear regressions derived from the recent xylem cation contents/elemental ratios (2010–2014; log_10_-transformed) and the present soil pH values (2012) as well as from historic xylem cation contents/ratios (1980–1984; log_10_-transformed) and corresponding soil pH values (1984) are listed in Table 2. As mentioned before, only those parameters for which bivariate correlations showed significant results in both years (1984 and 2012) were used as input variables for the stepwise regression. A comparison of historic and recent cation contents/elemental ratios and corresponding soil pH values in H_2_O and KCl extracts showed different results in the different soil depths and distances. In the topsoil of the stemflow area (S 0–5), no significant correlations of xylem contents/elemental ratios with soil pHs were found for both years. However, in the between trees area, these correlations were significant for both years for Mn in B 0–5 and B 30–40. Significant correlations for both years between xylem ratios and pH values were revealed for Ca/Mn, Mg/Mn, and Mn/Al in the top soil and in the deeper soil of the between trees area (B 0–5 and B 30–40). Out of these ratios, stepwise regression selected xylem Mg/Mn as the best predictor of soil pH. Correlation of the ratio of S 0–5/B 0–5 (i.e., soil pH value in the top soil of the stemflow area divided by that in the top soil of the between trees area) for both years in both soil extracts (H_2_O, KCl) yielded significant results only for Mg/Mn. Hence, the used regression equations, developed for different soil depths and distances from beech stems, were as follows (level of significance of adjusted *R*^2^ is shown as *p < 0.05; **p < 0.01; ***p < 0.001):

**Table 2:** Significant linear correlation coefficients between soil pH (H_2_O; KCl) measured in 2012 and logarithmic dendrochemical parameters of recent 5-year stemwood segments (2010–2014) and between soil pH (H_2_O; KCl) measured in 1984 and logarithmic dendrochemical parameters of historic 5-year stemwood segments (1980–1984) at 14 beech stands in the Vienna Woods, respectively. Parameters include all measured stemwood cations and the ratios Ca/Mg, Ca/Mn, Mg/Mn, and Mn/Al in the infiltration zone of stemflow near the base of the stem (S 0–5) and in the between trees area (B 0–5, B 30–40; given ranges are soil depths in cm) in 1984 and 2012. Level of significance is shown as ns: not significant; *p < 0.05; **p < 0.01; *N* = 14 stands

log(10)		S 0–5		B 0–5		B 30–40		S 0–5/B0–5	
		1984	2012	1984	2012	1984	2012	1984	2012
Ca	pH_H2O_	ns	ns	ns	ns	ns	ns	ns	ns
	pH_KCl_	ns	ns	ns	ns	ns	ns	ns	ns
Mg	pH_H2O_	ns	0.78**	ns	0.78**	ns	0.82**	ns	ns
	pH_KCl_	ns	0.70**	ns	0.69**	ns	0.67**	ns	ns
K	pH_H2O_	ns	0.58*	ns	0.64*	ns	0.61*	ns	ns
	pH_KCl_	ns	0.56*	ns	0.57*	ns	0.56*	ns	ns
Mn	pH_H2O_	ns	ns	− 0.70**	− 0.71**	− 0.79**	− 0.62*	0.59*	ns
	pH_KCl_	ns	ns	− 0.68**	0.73**	− 0.70**	− 0.73**	0.60*	0.62*
Fe	pH_H2O_	0.61*	ns	0.76**	ns	ns	0.55*	− 0.55*	ns
	pH_KCl_	ns	ns	0.76**	ns	0.60*	ns	− 0.62*	ns
Al	pH_H2O_	ns	ns	ns	ns	ns	ns	ns	ns
	pH_KCl_	ns	ns	ns	ns	ns	ns	ns	ns
Ca/Mg	pH_H2O_	ns	ns	ns	ns	ns	ns	ns	ns
	pH_KCl_	ns	− 0.58*	ns	ns	ns	ns	ns	ns
Ca/Mn	pH_H2O_	ns	ns	0.67**	0.64*	0.65*	0.55*	− 0.54*	ns
	pH_KCl_	ns	ns	0.65*	0.64*	0.54*	0.64*	− 0.54*	− 0.58*
Mg/Mn	pH_H2O_	ns	0.63*	0.75**	0.82**	0.83*	0.74**	− 0.63*	− 0.55*
	pH_KCl_	ns	0.61*	0.71**	0.81**	0.72**	0.81**	− 0.63*	− 0.64*
Mn/Al	pH_H2O_	ns	ns	− 0.59*	− 0.64*	− 0.66*	− 0.58*	ns	ns
	pH_KCl_	ns	ns	− 0.56*	− 0.64*	− 0.56*	− 0.67**	ns	0.58*

B 0–5

1 $$ \mathrm{pH}\;\left({\mathrm{H}}_2\mathrm{O}\right)=4.659+1.559\times \left[{\log}_{10}\left(\mathrm{Mg}/\mathrm{Mn}\right)\right]\kern1.25em \left(\mathrm{adj}.{R}^2=0.64\ast \ast \ast \right) $$

2 $$ \mathrm{pH}\left(\mathrm{KCl}\right)=3.263+1.789\times \left[{\log}_{10}\left(\mathrm{Mg}/\mathrm{Mn}\right)\right]\kern1.25em \left(\mathrm{adj}.{R}^2=0.63\ast \ast \ast \right) $$

B 30–40

3 $$ \mathrm{pH}\ \left({\mathrm{H}}_2\mathrm{O}\right)=4.881+1.734\times \left[{\log}_{10}\ \left(\mathrm{Mg}/\mathrm{Mn}\right)\right]\ \left(\mathrm{adj}.{R}^2={0.52}^{\ast \ast}\right) $$

4 $$ \mathrm{pH}\ \left(\mathrm{KCl}\right)=3.337+2.071\times \left[{\log}_{10}\ \left(\mathrm{Mg}/\mathrm{Mn}\right)\right]\ \left(\mathrm{adj}.{R}^2={0.62}^{\ast \ast \ast}\right) $$

S 0–5/B 0–5

5 $$ \mathrm{pH}\ \left({\mathrm{H}}_2\mathrm{O}\right)=0.957-0.105\times \left[{\log}_{10}\ \left(\mathrm{Mg}/\mathrm{Mn}\right)\right]\ \left(\mathrm{adj}.{R}^2={0.24}^{\ast}\right) $$

6 $$ \mathrm{pH}\ \left(\mathrm{KCl}\right)=0.980-0.187\times \left[{\log}_{10}\ \left(\mathrm{Mg}/\mathrm{Mn}\right)\right]\ \left(\mathrm{adj}.{R}^2={0.37}^{\ast \ast}\right) $$

**Fig. 8:**
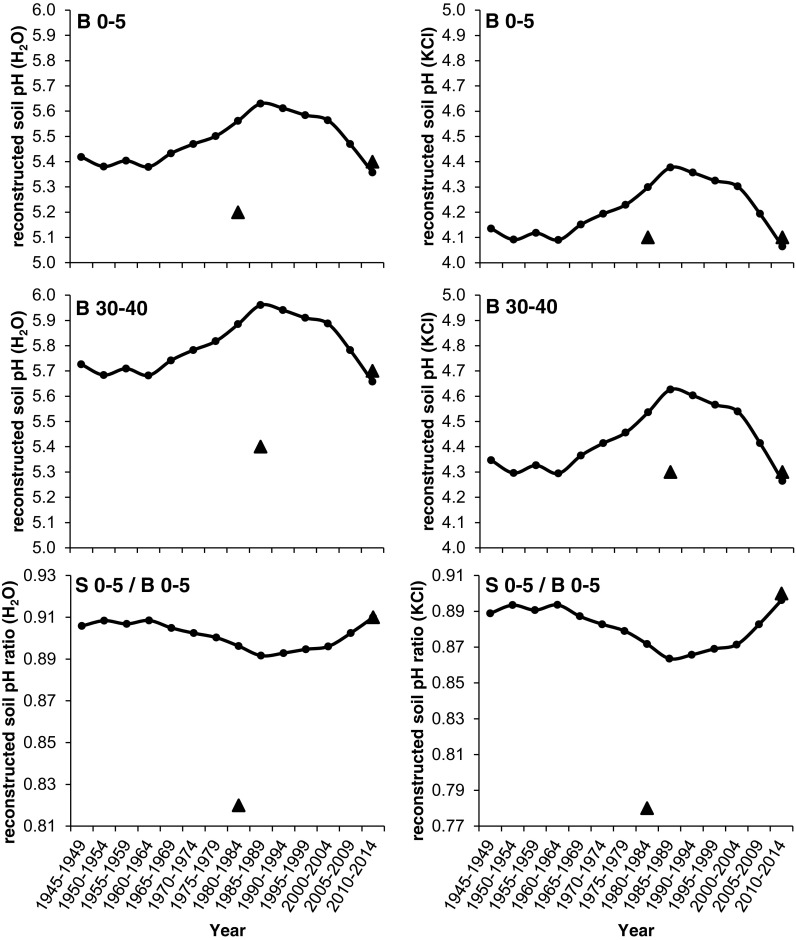
Mean reconstructed soil pH values in H_2_O and KCl extracts (1945–1949 to 2010–2014) at beech (*Fagus sylvatica*) stands in the Vienna Woods. Reconstructions are based on regressions of Mg/Mn ratio on soil pH in 0–5 and 30–40 cm depth in the between trees area (B 0–5 and B 30–40) and on soil pH ratio (S 0–5/B 0–5; i.e., soil pH in 0–5 cm depth in the stemflow area (S 0–5) divided by that in the between trees area (B 0–5)), respectively. Regression equations (1) to (6) are given in the text. The actual measured soil pH data in 1984 and 2012 (filled triangle) are given in Table 1 as well

Based on these equations, soil pH values were reconstructed over the last several decades at 0–5 and 30–40 cm soil depth in the between trees area of beech stands as well as at 0–5 cm soil depth for soil pH ratio S 0–5/B 0–5 (soil pH value in the stemflow area divided by that in the between trees area) and were plotted in Fig. 8. The mean measured soil pH values and soil pH ratios in 1984 and 2012 (see Table 1) of the beech stands were plotted in Fig. 8 as well for validating the models. The reconstructed soil pH values ranged from 5.36 to 5.63 (pH measured in H_2_O) and 4.06 to 4.38 (pH measured in KCl) in B 0–5 and from 5.66 to 5.96 (pH_H2O_) and 4.26 to 4.63 (pH_KCl_) in B 30–40, respectively. The reconstructed soil pHs and soil pH ratios failed to match the measured 1984 data. Reconstructed values even peaked, while actual pH values showed minimum values.

The results of linear regressions derived from the recent xylem cation contents/elemental ratios (2010–2014; log_10_-transformed) and the present exchangeable soil Ca contents (2012) as well as from historic xylem cation contents/ratios (1980–1984; log_10_-transformed) and corresponding exchangeable soil Ca contents (1984) are listed in Table 3. Significant correlations for both years between xylem cation contents and exchangeable soil Ca contents were revealed for Mn in the topsoil of the stemflow area (S 0–5) and K and Mn in the between trees area (B 0–5). Correlations between xylem ratios and exchangeable soil Ca contents yielded significant results for Ca/Mn, Mg/Mn, and Mn/Al in S 0–5 and B 0–5 for both years. In the deeper soil horizon of the between trees area (B 30–40), no significant correlations of xylem cation contents/elemental ratios with exchangeable soil Ca contents were found for both years. Again, stepwise regressions selected the xylem Mg/Mn ratio as best parameter for reconstructing exchangeable soil Ca. Hence, the used regression equations, developed for different soil depths and distances from beech stems, were as follows (level of significance of adjusted *R*^2^ is shown as *p < 0.05; **p < 0.01; ***p < 0.001):

**Table 3:** Significant linear correlation coefficients between exchangeable soil Ca (μg g^−**1**^) content measured in 2012 and logarithmic dendrochemical parameters of recent 5-year stemwood segments (2010–2014) and between soil exchangeable Ca (μg g^−**1**^) content measured in 1984 and logarithmic dendrochemical parameters of historic 5-year stemwood segments (1980–1984) at 14 beech stands in the Vienna Woods, respectively. Parameters include all measured stemwood cations and the ratios Ca/Mg, Ca/Mn, Mg/Mn, and Mn/Al in the infiltration zone of stemflow near the base of the stem (S 0–5) and in the between trees area (B 0–5, B 30–40; given ranges are soil depths in cm) in 1984 and 2012. Level of significance is shown as ns: not significant; *p < 0.05; **p < 0.01; *N* = 14 stands

log(10)	S 0–5		B 0–5		B 30–40	
	1984	2012	1984	2012	1984	2012
Ca	ns	ns	ns	ns	ns	ns
Mg	ns	0.65*	ns	0.67**	ns	0.60*
K	0.60*	ns	0.54*	0.54*	ns	0.58*
Mn	− 0.72**	− 0.62*	− 0.69**	− 0.73**	ns	− 0.63*
Fe	0.79**	ns	0.80**	ns	0.64*	ns
Al	ns	ns	ns	ns	ns	ns
Ca/Mg	ns	ns	ns	ns	ns	ns
Ca/Mn	0.72**	0.54*	0.66*	0.63*	ns	ns
Mg/Mn	0.73**	0.71**	0.70**	0.81**	ns	0.71**
Mn/Al	− 0.55*	− 0.53*	− 0.55*	− 0.64*	ns	− 0.54*

S 0–5

7 $$ \mathrm{Ca}\ \left[\upmu \mathrm{g}\ {\mathrm{g}}^{-1}\right]=132.694+1500.651\times \left[{\log}_{10}\ \left(\mathrm{Mg}/\mathrm{Mn}\right)\right]\ \left(\mathrm{adj}.{R}^2={0.46}^{\ast \ast}\right) $$

B 0–5

8 $$ \mathrm{Ca}\ \left[\upmu \mathrm{g}\ {\mathrm{g}}^{-1}\right]=48.841+3344.578\times \left[{\log}_{10}\ \left(\mathrm{Mg}/\mathrm{Mn}\right)\right]\ \left(\mathrm{adj}.{R}^2={0.62}^{\ast \ast \ast}\right) $$

**Fig. 9:**
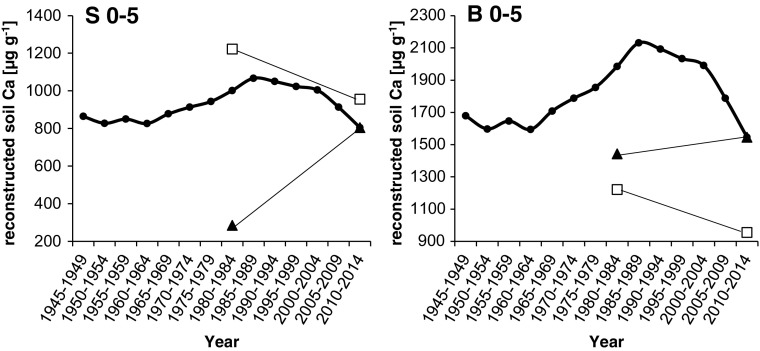
Mean reconstructed exchangeable soil Ca contents (μg g^−1^) from 1945–1949 to 2010–2014 at beech (*Fagus sylvatica*) stands in the Vienna Woods. Reconstructions are based on regressions of Mg/Mn ratio on soil Ca content in 0–5 cm depth in the stemflow area (S 0–5) and the between trees area (B 0–5). Regression equations (7) to (8) are given in the text. The actual measured Ca contents of soil (filled triangle) and of fresh beech foliage (open square; μg g^−1^ divided by 10) in the years 1984 and 2012, connected via a trend line, are given in Table 1 as well

Based on these equations, exchangeable soil Ca contents were reconstructed over the last several decades at 0–5 cm soil depth in the stemflow area (S 0–5) and the between trees area (B 0–5) of beech stands and were plotted in Fig. 9. The mean measured exchangeable soil Ca contents in 1984 and 2012 (see Table 1) at the beech stands were plotted in Fig. 9 as well for validating the models. The reconstructed soil Ca contents ranged from 804.7 to 1067.1 μg g^−1^ in S 0–5 and from 1546.5 to 2131.4 μg g^−1^ in B 0–5. Reconstructed exchangeable soil Ca contents did not match the measured values in 1984, showing similar patterns as the reconstructions of soil pH (compare Fig. 8).

### Foliage and Relationship Between Tree-Ring and Foliar Chemistry

Mean foliar macro nutrient contents at the 14 studied beech stands in 1984 and 2012 are presented in Table 1. While contents of N and S did not change significantly, contents of the base cations Ca, Mg, and K decreased markedly within the last three decades (see letters in Table 1).

Bivariate linear correlations between stemwood (log_10_-transformed) and foliar contents for the same element were significant and positive for Mn only (data not shown). However, again, element ratios give a much better perspective of how foliar contents are reflected in the chemistry of the transpiration stream due to normalizing for content fluctuations. Significant and positive correlations between the corresponding stemwood and foliar ratios were recorded for Ca/Mn and Mg/Mn for both years (1984 and 2012) and Mn/Al in 2012 (no foliar Al was measured in 1984). This kind of relation was strongest for the Mg/Mn ratio (*R* = 0.70, *p* < 0.01 in 1984; *R* = 0.83, *p* < 0.001 in 2012) supporting the choice of Mg/Mn tree-ring ratios for reconstructing soil pH.

Foliar contents of Ca, Mg, and K and corresponding ratios in 1984 and 2012 are plotted together with stemwood contents in Fig. 7, indicating similar changes over time. Reconstructed exchangeable soil Ca contents (Fig. 9) match nicely with Ca foliar trends between 1984 and 2012 as well.

## Discussion

### Will the Soils Within the Stemflow Area of Beech Approach the Same Chemical Status of the Between Trees Area After Removal of Stemflow?

Acidic deposition is considered a major cause for lowered soil pH in forest ecosystems. As acidic deposition levels have declined now, the question arises whether soils will recover from those acid inputs. Soil pH (KCl) and exchangeable Ca contents in beech stands increased in the top soil from 1984 to 2012 (Figs. 4 and 5; Table 1) at almost all sites which is in accordance with other studies in Europe (Jandl et al. 2012; Reininger et al. 2011) and North America (Lawrence et al. 2015). However, soil recovery (expressed as increase of soil pH and exchangeable Ca and Mg) was accelerated in the infiltration zone of stemflow compared to the between trees area (see Table 1). Increased inputs of bases into the stemflow area in the last years may not only be caused by weathering but also by the base cation pump effect of beech (Berger et al. 2006) as well as canopy leaching and associated deposition of base cations via stemflow (Berger et al. 2016). Despite distinct signs of soil recovery in the stemflow area (e.g., increased soil pH and higher contents of exchangeable Ca and Mg), pH (KCl) values and contents of exchangeable Ca in 2012 were still lower at about two thirds of the beech stands compared to the between trees area (Fig. 4) and a similar trend was found for Mg (Table 1). These findings are in line with Berger and Muras (2016) who hypothesized that the micro-spatial heterogeneity of soil columns downhill of a beech stem is a function of historic acid loads (stem area received much higher deposition loads in the past than the between trees area) and time (a space-for-time substitution due to higher soil solution fluxes close to the stem). When looking at the different soil depths in the between trees area, there was a clear trend of increasing pH values (H_2_O; KCl) with increasing soil depth in 2012, whereas mean Ca contents were lower in B 30–40 compared to the top soil (B 0–5; Table 1, Fig. 5) which was not consistent with the conceptual model of recovery that assumes replenishment of bases from weathering as cation leaching fluxes are reduced by declining acidic deposition. As stated in our previous study (Berger et al. 2016), this pattern was caused by mobilization of historic S and associated leaching of base cations with high amounts of sulfate, released within the last decades. Furthermore, we suggested in that study that the Ca pump of beech will alkalize the surface soil and acidify the subsoil from which Ca is drawn (Berger et al. 2016).

Similar changes of soil chemical properties were observed at the adjacent strip cut plots (2015) upon removal of stemflow water. Although pH (KCl) in the stemflow area increased at almost all strip cut plots (2015) compared to the beech stands in 2012 (Fig. 4), soils have either stabilized or slightly recovered in the top layers of the between trees area. The increase in pH (KCl) in S 0–5 is in agreement with the pH development observed by Falkengren-Grerup and Björk (1991) near beech stumps of different ages. As documented by Matschonat and Falkengren-Grerup (2000), after only 8 years of experimentally removing stemflow water of beech trees pH (KCl) and saturation with Ca^2+^ and Mg^2+^ increased close to the stem indicating that recovery probably reflected both the steady decline in acidic deposition over time and the removal of acidifying stemflow water. Mean soil pH values and contents of exchangeable Ca and Mg were still lower in the infiltration zone of stemflow after removal of beech stems compared to the between trees area; however, differences were very small and not significant (see letters in Table 1). Matschonat and Falkengren-Grerup (2000) proposed that the removal of stemflow water can serve as a model for the decrease in overall deposition and the observed recovery of pH and base saturation in the between trees area may be the effect of the ongoing decrease in acidic deposition. In accordance with this finding, our study showed an increase in mean pH (KCl) and exchangeable Ca and Mg from 2012 to 2015 not only in the top layers of the stemflow area (S 0–5: pH 3.55 vs. 4.22; Ca 0.80 vs. 1.96; Mg 0.08 vs. 0.16; units in mg g^−1^) but also in the between trees area (B 0–5: pH 4.06 vs. 4.30; Ca 1.55 vs. 2.00; Mg 0.13 vs. 0.14; units in mg g^−1^) after removal of beech stems, supporting the theory that changes in soil chemical properties may be a response to changes in the overall deposition regime. However, as proposed in a previous study (Berger et al. 2016), most soils acidify naturally and weathering did not keep pace with natural acidification pressures in times before air pollution, which is why a full recovery of soils from enhanced acidic deposition in the capacity sense may not be possible. In accordance with this scenario, it seems questionable that a full recovery will be achieved at all but the much higher reduction of acid loads in the stemflow area caused a quicker replenishment of base cations.

Finally, we have to give a complex answer to our research question 1: Even though soils recovered markedly in the top layers of the stemflow area from 1984 to 2012 in beech stands, they did not reach as favorable conditions as in the between trees area. In the between trees area and especially in deeper soil layers, recovery may be actually delayed. Although soil conditions improved even further after removing stemflow water by cutting beech trees, it is questionable that preindustrial soil pH and base cation levels for the whole soil profile will be reached within the next decades.

### Can the Rate of Reversibility of Soil Acidification Be Estimated Using Soil pH Changes as a Function of Time Since Felling?

Beech trees have long been known to alter physical and chemical properties of the soil related to the distance from the stem. Especially the stemflow-induced microsite around the tree often proved to have a lower pH (Falkengren-Grerup 1989; Chang and Matzner 2000; Matschonat and Falkengren-Grerup 2000), lower contents of exchangeable base cations (Berger et al. 2016; Lindebner 1990), and lower cation exchange capacity (Matschonat and Falkengren-Grerup 2000) compared to soils located further away from the stem. Hence, we analyzed pH changes along vertical slope gradients from beech stumps from different years of felling to assess the reversibility of acidified soils. The decreasing pH (KCl) difference between proximal and distal area from beech stumps in 10–20 cm soil depth from 10 to 25 years after the acidifying stemflow had ceased implies that the soil partly returned to the overall acidity of the stand (Fig. 6). However, a difference between proximal and distal soil pH still existed after 30 years and the small difference between the 15–20 year and 25–30 year classes indicates that a full recovery does not occur. Falkengren-Grerup and Björk (1991) observed a reduction in soil acidity by about 50% after felling of beech trees in southern Sweden that was most pronounced during the initial 15 years and little additional recovery was measured after that time. Application of a theoretical model on acidification and reversibility of whole catchments estimated the recovery of soil pH to be of the order of decades (Cosby 1987). However, due to beech stump decomposition, it is almost impossible to study reversibility much further back than 30 years (Falkengren-Grerup and Björk 1991). These results support the changes in the studied stemflow gradients, documenting initially large differences between proximal and distal areas but not reaching a full recovery within 30 years.

Surprisingly, no difference in pH (KCl) between S 10–20 and B 10–20 in the present study was found in the youngest year class (0–5 years since felling of beech trees). As mentioned before, mitigating factors like base cation pump of beech, canopy leaching, and associated deposition of base cations via stemflow may have caused increased inputs of bases into the stemflow area more recently which are considered important factors improving the condition of the soil within the microsites around trees. Berger et al. (2008) proposed that cation leaching via throughfall and stemflow at a beech stand on Flysch was primarily driven by organic anions and bicarbonate rather than by cation exchange reactions with H^+^ as deposition of acidifying substances declined.

In conclusion, we can answer our research question 2 that due to a decreased input of acidifying substances in the last decades, soil pH (KCl) close to the stem increased markedly and the longer the time since felling, the smaller became the difference between proximal and distal area. However, differences between the distances were still detectable after 30 years indicating that soils may not recover fully from acidification, or do so at a rather slow rate.

### Are Changes of Soil Chemical Properties over the Last Seven Decades Reflected in the Stemwood of Beech?

Radial distributions of elemental contents in tree rings do not only vary between elements, but also between tree species and soil characteristics. The availability of cation nutrients is often hindered by increased susceptibility to leaching or erosion losses in acidic soils or decreased solubility in more alkaline soils, respectively, and therefore the relation between soil and mineral element availability suggests a direct environmental control on wood resorption of elements (McCauley et al. 2009; Meerts 2002). Shortle et al. (1997) argued that leaching and cation mobilization via Acid Rain around the 1980s was initially characterized by increased cation contents in the stemwood followed by depletion in more acidic soils which is in agreement with the pattern of Mg stemwood content and elemental ratio of Mg/Mn in this study, which will be further discussed below.

In our study, general trends of declining cation stemwood contents were recorded only for K and Mg since the mid-1980s, respectively (Fig. 7). Penninckx et al. (2001) also found a decreasing pattern of K in beech and two coexisting species with contrasting wood structure which could point to a long-term process of soil acidification. However, decreasing cation contents from pith to cambium have also been commonly observed in other studies with various environmental contexts, which is why linking such outwardly decreasing content gradients to cation depletion in the soil is questionable (Hagemeyer et al. 1989; Momoshima et al. 1995; Riitters et al. 1991).

There are several factors that can account for the radial variations in xylem contents. One endogenous factor is the pattern of radial variation in living cells. In spite of the lack of a clearly differentiated sapwood, an increase in phosphorus and nitrogen in the last 10–15 years might still be an indication for a higher proportion of living cells in the outer wood (Penninckx et al. 1999, 2001). Like N and P, magnesium is an element usually associated with living cells and therefore would have been expected to increase towards the bark as well (De Visser 1992). The finding of a steady increase of Mg until the 1980s followed by a decrease in the last years strongly suggests an environmental influence. Such declining patterns of Mg in beech wood in the last decades were also confirmed by Penninckx et al. (1999) as well as by Bondietti et al. (1990) in red spruce trees in areas subject to strong acidic deposition. The variation pattern of Mg in this study might be ascribed to cation mobilization via Acid Rain followed by depletion through leaching as suggested by these authors. Watmough (1997) reported that changes of cation contents in younger xylem coincided with the hypothesized changes in chemistry of poorly buffered soils resulting from soil acidification due to accelerated acidic deposition. Soil acidification due to acidic deposition usually results in the mobilization of Mn because of its increase in availability with decreasing soil pH. Therefore, metals such as Mn have been used as reliable bioindicators of pH variations in the soil (Guyette et al. 1992). In the context of a forest ecosystem in a polluted area, the finding of an increase in Mn content since the early 1980s is in agreement with a hypothesis of soil acidification. In contrast, Kuang et al. (2008) and Penninckx et al. (1999) reported decreasing Mn contents in the xylem from pith to bark. They hypothesized that available Mn within rooting depth is being exhausted by long-term leaching because of a limiting pool of readily soluble Mn (Penninckx et al. 1999).

Jensen et al. (2014) showed a decrease in the Ca/Mn ratio in wood of *P. serotin*e and *L. tulipifera* over time due to experimental watershed acidification. They concluded that decreasing Ca/Mn ratios indicate a decline in Ca and an increase in Mn contents since base cation depletion is expected to occur together with increased Mn contents. This finding is in line with our results which showed a steady decrease of Ca/Mn with two main peaks in 1985–1989 and 1995–1999, indicating desorption of Ca from the soil exchanger via atmospheric acid anion inputs (Fig. 7).

Decreasing contents of K and Mg (more recently) as well as an increase in Mn content in beech stemwood may be interpreted according to the cited literature above as impact of atmospheric deposition of acidifying substances. However, decreasing Ca and particularly increasing Al contents in stemwood, often regarded as reliable indicators of soil acidification (Cutter and Guyette 1993; McLaughlin and Wimmer 1999), could not be confirmed in the present study. In addition, as stated above (compare Figs. 4 and 5; Table 1), soil data mainly indicated recovery from these acidic inputs of the early 1980s (e.g., increase of pH, Ca, and Mg). At this point, it must be pointed out that the soils on Flysch in the Vienna Woods, characterized by high cation exchange capacity and high base saturation (Muras 2012; Hanousek et al. 2017), are capable of buffering huge amounts of acid inputs. This means, high inputs of acid anions during the 1980s will cause equivalent leaching of base cations, since anion adsorption capacities are low for these high pH soils (Hanousek et al. 2017). Hence, we conclude that the following patterns of temporal stemwood contents clearly indicate mobilization of base cations via Acid Rain during the 1980s and early 1990s: maximum values for Mg, Ca, Ca/Mn, and Mg/Mn but minimum values for Ca/K and Ca/Mg (leaching occurs in the order of K > Mg > Ca according to the lyotropic series) during this period. However, the fact that stemwood contents were declining in recent times does not indicate that these cations have been exhausted but are simply the effect of a general decrease in ion concentration in the soil solution after acid anion input has ceased.

In agreement with Jonard et al. (2012), we suggest that the decrease in ion concentration in the soil solution caused decreased uptake rates of base cations in 2012. According to the mobile anion concept by Reuss and Johnson (1986), “the concentration of anions in solution will control the total concentrations of cations, while the composition of cations in solution should be controlled by equilibration with what is usually a large pool of cations adsorbed on soil particles.” Since acidic deposition declined from 1984 to 2012, we hypothesize that base cation concentrations in the soil solution of the studied soils, characterized by high base saturations, declined as well. The fact that declines from 1984 to 2012 are reflected both in stemwood as well as in foliage contents (Table 1; similar trend lines in Figs. 8 and 9) strongly supports this hypothesis.

However, in contrast to foliage parameters (see above), measured soil contents and ratios in the years 1984 and 2012 do not mimic the general trend of stemwood contents at all (see Fig. 7). None of the bivariate regressions between stemwood content and the corresponding exchangeable soil content of Ca, Mg, and K and their ratio combinations were significant, except for K in S 0–5 in both years (1984 and 2012). This finding further strengthens the stated hypothesis above.

Finally, we can answer our research question 3 that mainly changes of soil solution chemistry instead of soil chemical properties over time were reflected in cation stemwood contents of beech indicating mobilization of base cations during the early 80s followed by a steady decrease thereafter. Though we did not measure soil solution chemistry, higher foliar nutrient contents at lower soil nutrient status in 1984, but vice versa in 2012, strongly support this statement.

### Are Beech Xylem Contents or Their Ratios Related with Measured Soil pH Values or Soil Nutrient Contents?

Bivariate linear regressions were performed to see if there is a relationship between cation stemwood contents and actually observed soil pHs in beech stands (Table 2). In the stemflow area, none of the performed regressions yielded significant results for both years (1984 and 2012). However, in the between trees area, Mn stemwood contents were negatively correlated with soil pH (H_2_O and KCl) in all soil depths indicating that Mn contents increase with decreasing pH. These results are in compliance with others who stated that single cation contents in the xylem were significantly correlated with soil pH and therefore could be used as biomonitors for soil pH changes (Guyette and Cutter 1994; Guyette et al. 1992). According to Guyette et al. (1992), Mn is suitable for the reconstruction of soil chemistry changes due to its abundance in soil and sufficient quantities in xylem to be accurately detected. Manganese stemwood content has been proposed as reliable indicator of variations in soil pH. However, as described by Guyette et al. (1992), the use should be limited to soils which are well drained, a precondition, which is not always fulfilled for soils on Flysch, which are temporarily waterlogged.

Other remarkable indicators of soil pH were found in cation ratios. As stated by Bondietti et al. (1989), normalizing for concentration declines or other fluctuations by calculating element ratios should give a better perspective of how the chemistry of the transpiration stream has changed over time in a tree and allows for comparisons of trends in trees of different ages. Indeed, comparing the bivariate correlation results of Table 2 with each other shows that the Ca/Mn, Mg/Mn, and Mn/Al ratio yielded significant correlation coefficients in the between trees area for both years. When looking at the soil pH ratio (S 0–5/B 0–5; i.e., soil pH in S 0–5, divided by that in B 0–5), only Mg/Mn showed a significant result in both years for pH. Numerous past studies have proposed that the effects of acidic deposition on forest soils could be indicated by ratios of tree-ring elements, such as xylem Ca/Mn and Mg/Mn of sugar maple in North America (Houle et al. 2002; Kogelmann and Sharpe 2006) and of Masson pine in China (Kuang et al. 2008), which were widely applied indicators to monitor the temporal changes of soil acidity. DeWalle et al. (1999) suggested that sapwood ratios of Ca/Mn and Mg/Mn were more reliable than Ca/Al because of lower Al contents compared to Ca and Mg which coincided with other studies (Houle et al. 2002). In our study, no significant relationship was found between xylem Ca/Al and soil pH suggesting that ratios using Mn are better indices than ratios using Al. Indeed, aluminum tends to be excluded from plant uptake at the absorbing tip of the tree root due to increasing pH at the interface which leads to a decreasing aluminum availability for uptake (Smith and Shortle 1996).

The results justify the conclusion that tree-ring chemistry may be useful as indicator of soil chemistry. However, when comparing linear correlation coefficients from 1984 with those from 2012, none of the regressions yielded significant results in both years. In 2012, soil pH in all horizons was correlated with the base cations Mg and K in stemwood but not in 1984 at all (Table 2), suggesting that the relationship between wood and soil chemistry has changed over time. This fact may further indicate that after a sufficiently long period of lacking external acidic input (Acid Rain), soil solution chemistry in 2012 reflects the pool of cations adsorbed on the soil exchanger, but this was not the case in 1984 when Acid Rain increased ion soil solution concentrations.

Similar results were found for the relationship between exchangeable soil Ca contents and beech cation stemwood contents/their ratios (Table 3). Significant correlations between xylem ratios and exchangeable soil Ca contents were revealed for Ca/Mn, Mg/Mn, and Mn/Al in the topsoil of the stemflow area (S 0–5) and the between trees area (B 0–5) for both years. In 2012, exchangeable soil Ca contents were correlated with the cations Mg, K, and Mn in the stemflow area (S 0–5) and the between trees are (B 0–5 and B 30–40).

Finally, we can answer our research question 4 that bivariate correlations revealed a relationship between soil chemical properties and wood chemistry. However, results from different years indicate for certain cases that the relationship between wood and soil chemistry has changed over time, meaning, that a very important precondition for the use of dendrochemistry for reconstructing soil chemical parameters was not fulfilled.

### Is Dendrochemistry a Useful Tool for Reconstructing Soil pH and Soil Nutrient Changes in the Studied Beech Stands and Can the Data Be Verified by Measured Soil Parameters in 1984?

Soil pH values at 0–5 and 30–40 cm soil depth in the between trees area as well as the soil pH ratio (S 0–5/B 0–5; i.e., soil pH value in the top soil of the stemflow area divided by that in the between trees area) in H_2_O- and KCl extracts were reconstructed over the last 70 years in beech stands in the Vienna Woods (Fig. 8). It was expected that the trend lines show a general decline of soil pH with increasing stand age which is supported by other studies who documented declining soil pH reconstructions with increasing age of the stand (Berger et al. 2004; Chen et al. 2010). As suggested by Berger et al. (2004), base cations are built into the stand’s biomass and as a consequence gradually acidifying the soil from the beginning to the end of a rotation period, which was also documented by Neubauer (2000) for beech on Flysch by analyzing soil and stand data. However, reconstructed soil pH values in the topsoil and deeper soil layers of the between trees area (B 0–5 and B 30–40) started to increase in the 1960s until it peaked in mid-1980s and were clearly above the measured soil pH data in 1984 (Fig. 8).

Reconstructed soil pH ratio (S 0–5/B 0–5) showed a decreasing trend until the early 1990s and an increase afterwards suggesting that soil pH recovered markedly in the top layers of the stemflow area as acidic deposition levels have declined. In accordance with our previous study (Berger et al. 2016), soil recovery (expressed as increase of soil pH) was accelerated in the infiltration zone of stemflow compared to the between trees area from 1984 to 2012 which is demonstrated by the upward trend of reconstructed soil pH ratio in this study (Fig. 8).

Valid soil pH and nutrient reconstructions should be verified against actual observed soil data, but the lack of long-term historical monitoring data on soil chemistry often created difficulty in validating those reconstructions (Chen et al. 2010). However, our study is unique since soil data exist for both 1984 and 2012 for the studied beech stands. As shown in Fig. 8, none of the measured soil pH values in 1984 were actually on the reconstructed pH trend lines but much lower. Reconstructed exchangeable soil Ca contents showed similar trends with highest values in the 1980s but clearly lower measured data in 1984 (Fig. 9). Assuming that in the year 2012, after the decline of atmospheric acidic input the soil solution (intensity parameter) reached an equilibrium condition with the soil exchanger (capacity parameter), any external input of anions will increase cation solution concentrations. This is exactly what happened in 1984. For that reason, we postulate that our reconstructed soil contents do rather reflect soil solution chemistry than available soil nutrients stored. The fact that reconstructed soil Ca contents match with trend lines of Ca foliage contents (Fig. 9) supports this statement.

We can answer our research question 5 that although it was possible to reconstruct soil pH and nutrients using dendrochemical methods, their validity could not be verified by measured soil pHs and nutrients from the 1980s for the studied beech stands. However, patterns of stemwood contents indicated times of increased mobilization of base cations via Acid Rain in the 1980s and consequently our reconstructions mimicked soil solution rather than soil exchanger chemistry.

## Conclusions

Fourteen beech stands in the Vienna Woods were analyzed in 1984 and 2012 to study how soil chemistry changed as acidic deposition decreased over the last three decades. Soil analyses indicated a recovery in the top layer of the stemflow area but in the between trees areas and especially in deeper soil horizons recovery may be delayed.

In addition, reversibility of soil acidification was evaluated, using strip cut plots of the same sites providing stumps from different years of felling, representing the years when acidic stemflow ceased to affect the soil. Although soil pH (KCl) in the stemflow area were higher at the strip cut plots compared to the adjacent beech stands, stemflow gradients still existed. Differences in soil pH (KCl) between proximal and distal area from the stumps were detectable even after 30 years indicating that soils may not recover fully from acidification, or if so, at a rather slow rate.

It was hypothesized that changes of soil chemical properties over the last seven decades are reflected in the stemwood of beech. In fact, patterns of stemwood contents of beech clearly indicated mobilization of base cations during the early 80s followed by a steady decrease thereafter. Though we did not measure soil solution chemistry, higher foliar nutrient contents at lower soil nutrient status in 1984, but vice versa in 2012, strongly support this statement. It was possible to perform reconstructions of soil pHs and nutrients using dendrochemical methods, but their validity could not be verified by measured soil pHs and nutrients in the 1980s for the studied beech stands. Hence, dendrochemical soil reconstructions must be interpreted with caution.

We conclude that patterns of stemwood contents indicated times of increased mobilization of base cations via Acid Rain in the 1980s and consequently our reconstructions mimicked soil solution rather than soil exchanger chemistry. Our conclusion provides a new hypothesis, worth to be tested: based upon the equilibrium state between soil solution and soil exchanger in recent years, soil solution and foliar chemistry can be reconstructed by dendrochemical methods.

In addition, our conclusions should be tested for other tree species with a sharper heartwood/sapwood transition than beech, which might be more suitable for detecting environmental changes in the soil.
